# The Impact of Ambient Temperature on Childhood HFMD Incidence in Inland and Coastal Area: A Two-City Study in Shandong Province, China

**DOI:** 10.3390/ijerph120808691

**Published:** 2015-07-23

**Authors:** Lin Zhu, Zhongshang Yuan, Xianjun Wang, Jie Li, Lu Wang, Yunxia Liu, Fuzhong Xue, Yanxun Liu

**Affiliations:** 1 Department of Epidemiology and Biostatistics, School of Public Health, Shandong University, Jinan 250012, China; E-Mails: zhu.lin@outlook.com (L.Z.); yuanzhongshang@sdu.edu.cn (Z.Y.); lijiesdu0816@163.com (J.L.); lulu_stat@hotmail.com (L.W.); yunxialiu@163.com (Y.L.); xuefzh@sdu.edu.cn (F.X.); 2 Shandong Center for Disease Control and Prevention, Jinan 250012, China; E-Mail: xjwang62@163.com

**Keywords:** temperature-disease association, distributed lag non-linear models, hand, foot and mouth disease, children, geographical heterogeneity

## Abstract

Hand, foot and mouth disease (HFMD) has been a substantial burden throughout the Asia-Pacific countries over the past decades. For the purposes of disease prevention and climate change health impact assessment, it is important to understand the temperature–disease association for HFMD in different geographical locations. This study aims to assess the impact of temperature on HFMD incidence in an inland city and a coastal city and investigate the heterogeneity of temperature–disease associations. Daily morbidity data and meteorological variables of the study areas were collected for the period from 2007 to 2012. A total of 108,377 HFMD cases were included in this study. A distributed lag non-linear model (DLNM) with Poisson distribution was used to examine the nonlinear lagged effects of daily mean temperature on HFMD incidence. After controlling potential confounders, temperature showed significant association with HFMD incidence and the two cities demonstrated different impact modes (*I^2^* = 96.1%; *p* < 0.01). The results highlight the effect of temperature on HFMD incidence and the impact pattern may be modified by geographical localities. Our findings can be a practical reference for the early warning and intervention strategies of HFMD.

## 1. Introduction

Hand, foot and mouth disease (HFMD) is a viral infection caused by a group of enteroviruses, mainly coxsackievirus A16 (CA16) and enterovirus 71 (EV71) [1,2]. The disease is characterized by a distinct clinical presentation of fever, or vesicular exanthema on the hands, feet, mouth, or buttocks [3]. While not usually fatal, serious complications can result from infection such as meningitis and encephalitis, which can lead to death [4]. Infants and children younger than 5 are more likely to acquire the infection [1,5]. HFMD is highly contagious and is transmitted by nasopharyngeal secretions such as saliva or nasal mucus, by direct contact, or by fecal–oral transmission. Infected persons are most contagious during the first week of the illness, but the period of communicability can last for several weeks. Many large outbreaks of HFMD occurred in Asia-Pacific countries over the past decades [6,7,8,9]. As one of the most serious epidemic areas, China was burdened with the control and prevention of HFMD. It was estimated that the number of patients with neurological or cardiopulmonary complications was 16,500 each year in China, and among these severe cases, the fatality rate was about 3.0% [1]. However, no effective vaccine or curative treatment is available yet, thus it is of great importance to build an early warning system. 

Global warming is currently affecting and will increasingly influence human life. Growing interest has been shown in assessing the impact of temperature on disease, especially infectious disease [10,11,12,13]. Strong epidemiologic evidence suggested that HFMD is climate sensitive; a relationship between ambient temperature and HFMD incidence has been documented in several studies, but the findings are not consistent [14,15,16,17,18]. Some studies revealed that temperature was positively related to the occurrence of HFMD. For example, a study in Singapore showed that every 1 °C increase in temperature above 32 °C elevated the HFMD incidence by 36% [18]. Research in Guangzhou, China and Fukuoka, Japan illustrated that the increase of HFMD incidence effect was 1.8% and 11.2%, respectively, per 1 °C increase of temperature [14,17]. Later research in Shanxi Province also supported a positive association [19]. Another study in Tokyo, Japan found a negative association between temperature and HFMD when the average temperature is above 25 °C and a decreasing trend also appeared above 26 °C in a study in Beijing [15,20]. The observed variance suggested that the effect of temperature may be modified by different geographical factors. Despite this speculation, comparative studies of associations between temperature and HFMD incidence in different areas are largely lacking. 

**Figure 1:**
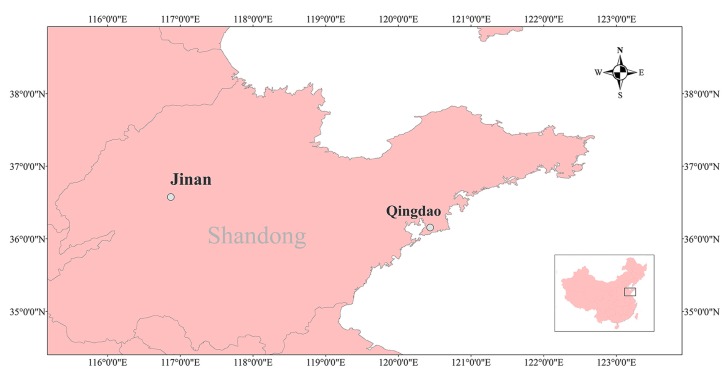
The geographical location of Jinan and Qingdao in Shandong province in China (map created with ArcGIS software, 9.3).

The objective of this study was to assess the impact of temperature on HFMD incidence in the two cities and, further, to identify the heterogeneity of temperature–disease association across the island and coastal locations. For this purpose, we conducted a time-series analysis of the association of daily HFMD incidence with meteorological variables from 2007 to 2012 in an inland city, Jinan (36°40′ N 116°59′ E), and a coastal city, Qingdao (36°04′ N 120°23′ E), in Shandong Province, China. The geographical location of the study area is shown in Figure 1. Jinan is the capital of Shandong Province, and Qingdao is one of the most important harbors in China. Both cities suffered severe and widespread epidemic of HFMD in recent years, and the epidemic features among the cities within the province were not homogenous. A better understanding of the effect of temperature on HFMD incidence would help in identifying high-risk periods and appropriate allocation of health care. Moreover, the results can provide scientific evidence for the early warning system and intervention strategies for HFMD.

## 2. Materials and Methods

### 2.1. Data Collection 

The daily morbidity data of HFMD from 1 January 2007 to 1 December 2012 were obtained from the China Information System for Disease Control and Prevention (CISDCP, http://www.cdpc.chinacdc.cn), including the basic social demographic characteristics of HFMD cases, and the pathogen type (CA16, EV71, and other EV) of some laboratory-confirmed cases. Laboratory evidence of enterovirus infection was detected by real-time PCR. The diagnosis of HFMD was made according to the clinical criteria established in the HFMD Control and Prevention Guide published by the Chinese Ministry of Health [21]. The case report cards were filled out by professional doctors, collected by trained reporters, and then input into the CISDCP within 24 h. In this paper, we mainly focused on child HFMD cases (0–5 years), which accounted for the majority of the total cases (described in the results section) and were particularly vulnerable to the temperature. The corresponding demographic data on Jinan and Qingdao between 2007 and 2012 were obtained from the Shandong Statistical Yearbook. Data on daily meteorological variables, including temperature, relative humidity, rainfall, and sunshine duration, were collected from the China Meteorological Data Sharing Service System.

### 2.2. Statistical Analysis

A quasi-Poisson generalized linear regression model combined with a distributed lag non-linear model (DLNM) was used daily to quantify the effect of temperature on reported incidence of child HFMD cases. The modeling framework is based on the definition of a cross-basis, a bi-dimensional space of functions specifying the dependency along the space of the predictor and along lags. The cross-basis functions are built combining the basis functions for the two dimensions, produced by applying existing or user-defined functions such as splines, polynomials, linear threshold, or indicators. Therefore, DLNM is a flexible model to simultaneously describe a non-linear exposure–response relationship and delayed effect [22]. The model was specified as:

*Log*[*E*(*Y_t_*)] = *α* + *βTemp_t,l_* + *NS*(*Hum_t_*,3) + *NS*(*Sun_t_*,3) + *NS*(*Time*,7/*year*) + γ*Dow_t_* + *vHoliday_t_*        , (1)

where *Y_t_* is the reported daily childhood HFMD incidence at day *t* (*t*=1, 2, 3, 4, …, 2192), *α* is the model intercept, *Temp_t,l_* is a matrix obtained by DLNM to model non-linear and distributed lag effects of temperature over the current day to lag *l* days, and *β* is the vector of coefficients for *Temp_t,l_*. A lag of 14 days was used to quantify the lagged effect of temperature. The mean daily temperature of the two cities (13.8 °C) was used as the reference value to calculate the relative risk (RR). Potential confounders, including the relative humidity (Hum), rainfall (Rain) and sunshine duration (Sun), were modeled as a natural spline with three degrees of freedom (*df*). Akaike’s Information Criterion for quasi-Poisson (QAIC) was adopted to choose the *df* for temperature. The final composition of the function was a natural cubic spline of temperature with four *df* and a natural cubic spline with four *df* for lag days. Furthermore, the day of the week (DOW) and public holidays (PH) were also included in the model to adjust for any deviation from the weekly pattern including public holidays. Season trend and long-term trends were controlled through a natural cubic spline with 7 *df* each year for time. We performed sensitivity analyses to assess the robustness of results by varying the *df* for time. 

To test the heterogeneity of effects across the two cities, the association was reduced to the overall relationship, cumulating the risk over the lag period. The heterogeneity was tested through multivariate meta-analysis and then reported by the multivariate extension of Cochran Q test and *I^2^* statistic [23]. To further compare the impact pattern of temperature on HFMD incidence between these two cities, we fitted the cubic polynomial regression of temperature on relative risk in each city given the non-linear association between temperature and HFMD incidence. A two-group *U*-test was then adopted to compare the corresponding regression coefficients obtained from these two polynomial regressions. 

All statistical analyses were conducted using R software (version 3.1.0), with the “dlnm” package to fit the distributed lag non-linear model and the “mvmeta” package to conduct multivariate meta-analysis. All statistical tests were two-sided in this study. *p* values of less than 0.05 were considered as statistically significant. 

## 3. Results

### 3.1. Descriptive Statistics 

In total there were 44,573 and 55,176 child (0–5 years old) HFMD cases reported in Jinan and Qingdao, respectively, from 2007 to 2012. The annual average child incidence rate of HFMD was 197 and 217 per 10,000, respectively, much higher than the annual average child incidence rate of HFMD in Shandong during the same period (144 per 10,000).

**Table 1:** Basic demographic characteristics of HFMD cases and meteorological data in Jinan and Qingdao, 2007–2012.

**Total Cases**	**Jinan**	**Qingdao**
	47,705	60,672
**Age**		
0–5 years	44,573 (93.4%)	55,176 (91.0%)
>5 years	3132 (6.6%)	5496 (9.0%)
**Sex (0–5 years)**		
Male	26,967 (60.5%)	33,989 (61.6%)
Female	17,606 (39.5%)	21,187 (38.4%)
**Pathogen (0–5 years)**		
Cox A16	699 (36.6%)	330 (27.4%)
EV71	830 (43.5%)	375 (31.1%)
Other EV	380 (19.9%)	501 (43.2%)
**Meteorological Variables**		
**Mean Temp (°C)**		
Mean ± SD	14.5 ± 10.6	13.0 ± 9.3
Min.	−10.0	−8.8
Median	16.3	13.1
Max.	35.0	29.0
**Mean Relative Humidity (%)**		
Mean ± SD	56.0 ± 20.7	69.9 ± 17.3
Min.	13.0	22.0
Median	55.0	71
Max.	100.0	100
**Rainfall (mm)**		
Mean ± SD	2.0 ± 8.1	2.3 ± 10.7
Min.	0	0
Median	0	0
Max.	143.8	241.2
**Sunshine Duration (h)**		
Mean ± SD	5.7 ± 3.9	5.9 ± 3.9
Min.	0	0
Median	6.5	6.9
Max.	13.5	12.8

Table 1 summarizes some basic demographic characteristics of HFMD cases from 2007 to 2012. It shows that the 0–5 age group constituted the majority of the victims in the outbreaks, accounting for 93.4% and 91.0% of all reported cases in Jinan and Qingdao, respectively. Thus, we mainly focused on the 0–5 age group in the time series analysis. Of 44,573 child HFMD cases in Jinan, 26,967 were boys and 17,606 were girls; of 55,176 HFMD cases in Qingdao, 33,989 were boys and 21,187 were girls. The male-to-female sex ratio was 1.54 and 1.60, respectively. Among 1909 and 1206 genotyped cases verified in the laboratory, EV71 and other EV were the predominant pathogens in Jinan and Qingdao. Jinan is characterized by a temperate continental monsoon climate and the average annual temperature is 14.5 °C. Qingdao belongs to the temperate maritime monsoon climate zone and the average annual temperature is 13.0 °C.

**Figure 2:**
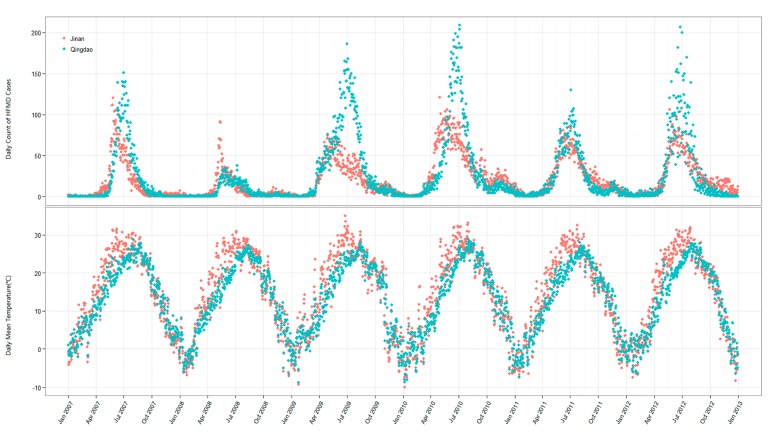
The daily distribution of HFMD incidence and mean temperature in Jinan and Qingdao, 2007–2012 (unit: incidence (per 100,000), temperature (°C)).

Figure 2 illustrates the daily distribution of HFMD incidence and mean temperature. The results indicate that the occurrence of HFMD presents significant seasonality in Jinan and Qingdao. It could be found that the incidence peak appeared in May and July in Jinan, and the epidemic peak in Qingdao was observed in July and June during the study periods. 

### 3.2. Temperature Lag–HFMD Incidence Association

**Figure 3:**
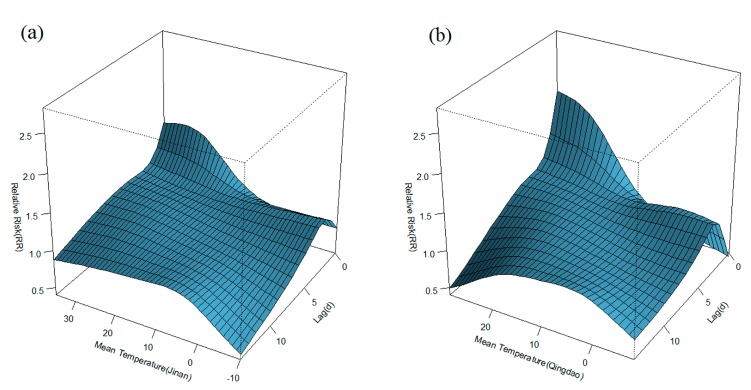
Relative risks of daily HFMD by daily mean temperature along 14 lag days in Jinan and Qingdao, adjusting for relative humidity, rainfall, sunshine duration, DOW, holidays, seasonal trend, and long trend: (**a**) Jinan, (**b**) Qingdao).

**Figure 4:**
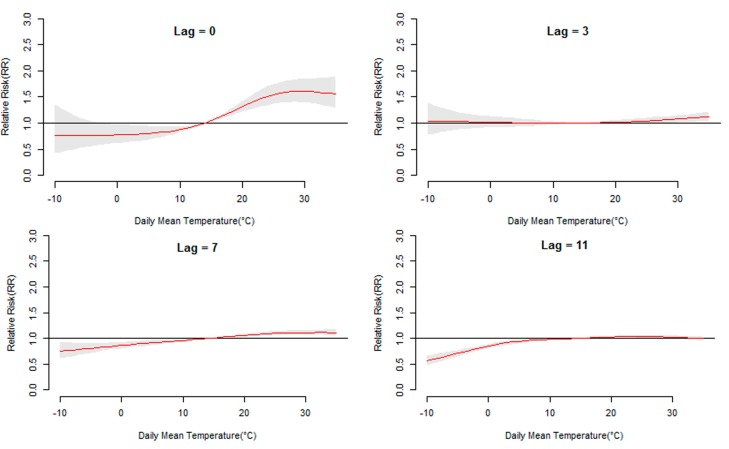

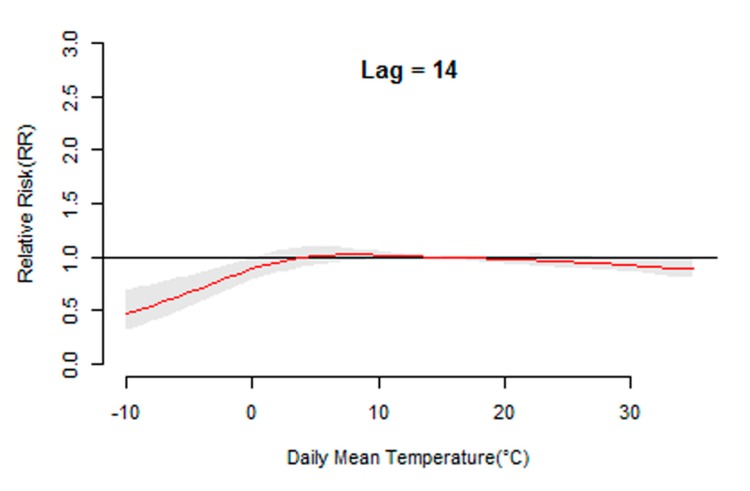
The relative risk of HFMD by daily mean temperature at a specific lag day (0, 3, 7, 11, and 14 days) in Jinan. The maximum likelihood estimate of RRs is shown as smooth red lines and the pointwise 95% confidence intervals are shown in the gray regions.

**Figure 5:**
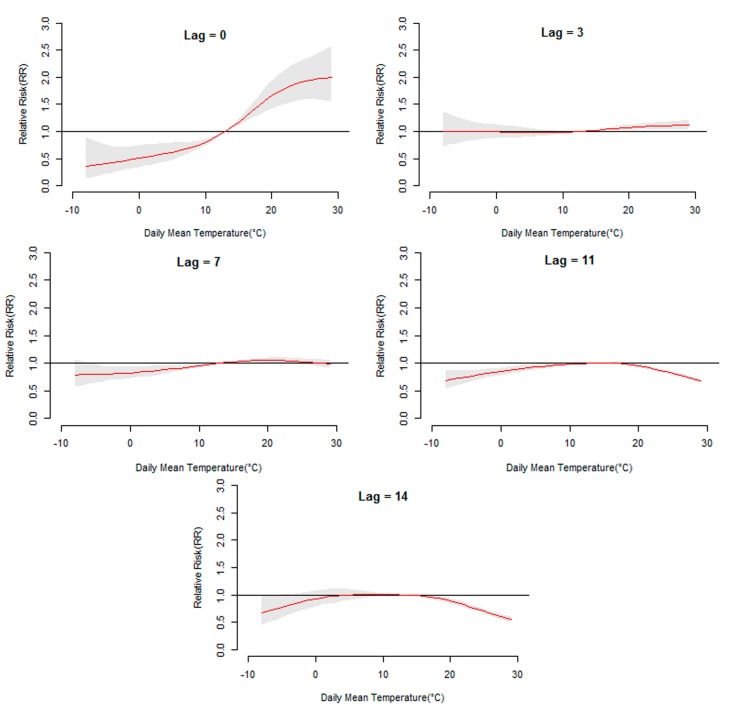
The relative risk of HFMD by daily mean temperature at a specific lag day (0, 3, 7, 11, and 14 days) in Qingdao. The maximum likelihood estimate of RRs is shown as smooth red lines and the pointwise 95% confidence intervals are shown in the gray regions.

Figure 3 displays the general pattern of relative risks (RR), as a function of temperature and lag, by showing three-dimensional plots of RR along temperature and 14 lag days. Overall, the estimated effects of temperature on HFMD incidence were non-linear, with higher relative risks at hotter temperatures. Detailed lag structures for temperature effects at a specific lag day (0, 3, 7,11, and 14 day) were shown in Figure 4 (Jinan) and Figure 5 (Qingdao). It was found that the high temperature had acute and short-term effects and then declined rapidly along the lag days. In addition, the high temperature effects in Qingdao appeared to be stronger and then diminished faster, while in Jinan the high temperature effects persisted longer.

### 3.3. The 14-Day Cumulative RR for Daily Mean Temperature

**Figure 6:**
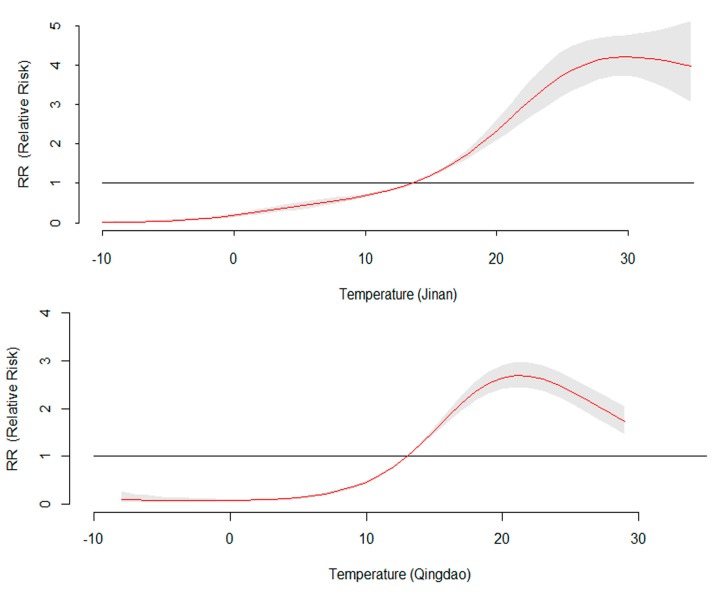
Effect of daily mean temperature on daily HFMD incidence over lags from 0 to 14 days in the study cities, controlling for relative humidity, rainfall, sunshine duration, DOW, holidays, seasonal trend, and long trend. The maximum likelihood estimate of RRs is shown as smooth red lines and the pointwise 95% confidence intervals are shown in the gray regions.

Figure 6 demonstrates the overall relationship between HFMD and temperature, showing the relative risk over the whole lag period. In Jinan, temperature generally showed a positive association with the HFMD incidence, achieving the maximum risk at 30 °C and then maintaining a high level. The highest RR value was 4.21 (95% CI: 3.74, 4.67). In Qingdao, although HFMD incidence increased with temperature at first, a negative association between temperature and HFMD was found when the temperature was above 21 °C. The peak RR value was 2.97 (95% CI: 2.75, 3.12). Sensitivity analyses varying the degrees of freedom (*df*) for time from six to eight per year indicated that the estimated effects did not change substantially. 

### 3.4. Heterogeneity Analysis

Multivariate Cochran Q-test was used to test the heterogeneity; the I-square statistic was up to 96.1% (Q = 102.6, *p*-value < 0.001). The result reveals the modification effect of geographical locations on temperature–disease association. Table 2 illustrates the location-specific association pattern between temperature and HFMD incidence. A significant difference could be found in quadratic terms (*p* < 0.0001) and cubic terms (*p* < 0.0001) between these two cities, while no significance emerged in linear term. 

**Table 2:** The location-specific association pattern between temperature and HFMD incidence.

Coefficients	Jinan	Qingdao	*p*
constant	−0.0613	−0.2727	0.0826
linear term	0.0336	0.0271	0.6777
quadratic term	0.0066	0.0149	<0.0001 *
cubic term	−0.0001	−0.0004	<0.0001 *
***R^2^***	96.89%	93.41%	

* statistically significant.

## 4. Discussion

Hand, foot and mouth disease (HFMD) has been a substantial burden throughout the Asia-Pacific countries over the past decades. Limited numbers of studies have examined the effects of temperature on HFMD incidence. However, comparative studies across different areas are largely lacking. We conducted a time-series study to examine the effect of temperatures on HFMD incidence in a coastal city and an inland city. To our best knowledge, this is the first study in China to assess the relationship between temperature and HFMD incidence in different areas using a distributed lag non-linear model (DLNM) on a daily basis, including properly evaluating the nonlinear associations and cumulative risks related to temperatures for different lag days. 

The two regions in this study demonstrated different temperature impact patterns. The effect of temperature on HFMD incidence in Jinan generally showed a positive relationship; the pattern was similar to the results of research performed in Singapore, Hong Kong, and Guangzhou [16]. However, the decreasing trend above 21 °C found in Qingdao was not consistent with these studies, whereas it echoed the findings in Tokyo and Beijing. Several reasons besides geographical location may cause the different patterns of temperature–incidence association. From the aspect of methodology, the adoption of different models can lead to variance. Our results highlighted the fact that the nonlinear association between temperature and HFMD cannot be ignored, and simple linear regression was undoubtedly insufficient to capture it. Compared with the generalized linear model (GLM) and the generalized additive model (GAM), the DLNM adopted in this study is a more biologically plausible method that provides a detailed representation of the non-linear exposure–response relationship. In addition, this method can avoid co-linearity problems among lagging exposure variables [22,24]. It has been frequently used to assess the effect of meteorological variables on disease. In previous research, various temperature indicators were employed to evaluate the association between temperature and HFMD, for example, maximum temperature, minimum temperature, mean temperature, and temperature difference. Though each indictor has its special mean, in this study we prefer to use daily mean temperature as a more accurate predictor, for it goes through the entire day and night, whereas maximum or minimum indictor only reflects the exposure for a short period [25,26].In addition, the selection of temporal scale could also influence the results. Previous studies mainly aggregated meteorological variables and incidence data by week or even month, which was not sensitive and specific enough to inform early warning systems. The detailed scale can provide more accurate information on the occurrence of childhood HFMD followed by the impending temperature [27]. From the standpoint of causative infectious agent, different predominant viruses may lead to different temperature impact patterns. For different predominant viruses, the suitable temperature conditions for growth, multiplication, and spread are not alike, nor is their tolerance for thermal effect [2]. In terms of population, diverse socioeconomic and medical standards, as well as different disease prevention and control policies, can also influence the outcome of temperature–disease association assessment [28,29]. 

Epidemiologically, temperature plays a fundamental role in the transmission of infectious diseases, and the impact is multifaceted. Temperature–disease associations are driven by a complicated interaction of pathogens, host population, and environmental factors [30,31,32]. A laboratory study found that thermal effect plays an important role on the infectivity of enterovirus [33]. Within a certain range, the higher the temperature is, the quicker the virus reproduces, and thus the fewer times it needs to be transmitted among the population. In addition, outdoor activity levels decrease during the cold seasons and increase when the temperature goes up. Thus temperature is related with the behaviors of the host population, which coincides with the probability of infection [34,35]. A previous study pointed out that the residents who live in coastal cities are more likely to take physical exercise than those who live inland [36]. High frequency of outdoor activities in public places could increase the risk of being infected. Qingdao is densely populated and attracts a large number of tourists every year, especially in the summer. A few HFMD cases may trigger an epidemic, or even an outbreak. This could potentially explain the acute and strong effect of high temperature in the coastal city. Another laboratory-based study indicated that enterovirus can survive well in a watery environment; we therefore speculate that more rainy days and higher humidity may exacerbate the epidemic of HFMD in coastal areas [37]. Although the exact mechanism that causes the heterogeneity across different geographical locations cannot be explained clearly, several factors including, but not limited to, the defenses and immunity of host population, and the infectivity and stability of pathogens, as well as other environmental factors, contribute to the different temperature–disease association [38].

Some limitations of this study should be acknowledged. First, results from one coastal and one island city may not be generalizable to other locations. More multi-city studies in diverse geographical areas are needed for further research. Second, surveillance data for HFMD do not capture all cases in the study area. The degree of under-reporting also varies in non-epidemic and epidemic periods [39,40]. Third, modifications from other socioeconomic factors, such as marital status, household income, and the use of heating or air conditioning, were not taken into consideration because of the lack of detailed individual information. 

## 5. Conclusions

In conclusion, our study revealed the non-linear relationship between temperature and childhood HFMD in coastal and inland regions. The results suggested that different patterns of temperature–disease association should be considered when formulating and optimizing public health intervention strategies. Future studies are required for more detailed understanding of the spreading mechanism of infection in certain geographical locations, which will be of critical importance to best prevent disease in those particular areas.
